# A spatiotemporal analysis of cattle herd movement in relation to drinking-water sources: implications for *Cryptosporidium* control in rural Kenya

**DOI:** 10.1007/s11356-021-17888-3

**Published:** 2022-01-17

**Authors:** Jessica R. Floyd, Emmah Kwoba, Thumbi Mwangi, Joseph Okotto-Okotto, Peggy Wanza, Nicola Wardrop, Weiyu Yu, Jim A. Wright

**Affiliations:** 1 School of Geography and Environmental Science, University of Southampton, Building 44, Highfield, Southampton, SO17 1BJ UK; 2 Centre for Global Health Research, Kenya Medical Research Institute, P.O. Box 1578-1400, Kisian campus, Kisumu-Busia Highway, Kisumu, Kenya; 3 Victoria Institute for Research on Environment and Development (VIRED) International, P.O. Box 6423-40103, off Nairobi Road, Rabuor, Kenya

**Keywords:** Cryptosporidium, Landscape, Kenya, Livestock, Animal movement, Water contamination

## Abstract

Given the increasing evidence that domestic contact with livestock is a risk factor for child diarrhoea in low- and middle-income countries, there have been calls for greater quantification of human-livestock contact in such countries. This study aimed to quantify seasonality in cattle proximity to domestic water sources and household compounds and develop a preliminary landscape model of faecal deposition by cattle. A total of 120 cattle in smallholder herds in the Asembo area of Siaya County, Kenya, were tracked over 1 week in April 2018 to July 2018 and November 2018 to February 2019 using GPS tracking devices. Dung deposition and behaviour were observed among 33 cattle from these herds over 185.4 hours. Mean cattle home ranges were small at 3.78 km^2^ and 5.85 km^2^ in the wet and dry seasons, respectively. There were significant differences between seasons in home range size, distance travelled from the household, and time spent tethered, but not in the time spent at domestic water sources or home range overlap with other herds. On average, 0.76 dung deposition events/hour were observed, with higher frequency in bulls. Variation in cattle proximity to household compounds and water sources did not account for seasonal variation in child diarrhoea in this population. The preliminary landscape model of faecal deposition by cattle could be further developed to inform interventions for safe separation of livestock and people, such as fencing and separate water troughs.

**Supplementary Information:**

The online version contains supplementary material available at 10.1007/s11356-021-17888-3.

## Introduction

Diarrhoeal disease is the second highest cause of death in children under 5 worldwide, killing over half a million under-fives each year (“WHO | Diarrhoeal disease,” 2017). Since several common diarrhoea pathogens (e.g. *campylobacter*, *salmonella*, and *Cryptosporidium* spp.) are harboured by animals as well as humans, there is growing evidence implicating livestock in diarrhoea transmission. Systematic review evidence (Zambrano et al. 2014) found a positive association in almost all included studies examining pathogen-specific diarrhoea in relation to animal husbandry-related risk factors. This has led to calls for greater understanding of potential transmission pathways via soil, hands, flies, fomites (i.e. objects such as utensils or toys likely to carry infection (Penakalapati et al. 2017)), and fluids including from water sources.

Among diarrhoeal pathogens infecting humans and livestock, the genera *Cryptosporidium* are common enteric parasites that cause significant morbidity and mortality via a diarrhoeal infection known as cryptosporidiosis (Checkley et al. 2015). The Global Enteric Multicentre Study (GEMS) identified *Cryptosporidium* as one of four pathogens to which most cases of moderate to severe diarrhoea (MSD) were attributable (Kotloff et al. 2013). *Cryptosporidium* transmission occurs through shedding of parasite oocysts in host faeces, which are immediately infective and can be ingested by other hosts through contaminated food and water. The infective dose is so low that just one oocyst is enough to cause an infection, and they are remarkably resistant to degradation: oocysts can survive for as long as 6 months suspended in water, and are also resistant to common chemicals used in water treatment (Smith et al. 2006). Moreover, some species like *C. parvum* can infect multiple host species, including humans and livestock, and have been responsible for previous waterborne outbreaks. This includes the largest waterborne disease outbreak in US history, which caused 403,000 cases in Milwaukee in 1993 (Kramer et al. 1996) as well as other outbreaks related to drinking water contamination (Glaberman et al. 2002). Although it can be difficult to prove the original source of an outbreak, water contamination by domesticated animals or livestock is widely recognised as a significant public health hazard (Graczyk et al. 2000). Thus, livestock are known to be a source of both direct infection and environmental contamination.

These qualities make elimination of *Cryptosporidium* oocysts in the environment very difficult, and with research into a vaccine still ongoing (Checkley et al. 2015; Ryan and Hijjawi 2015), evidence is needed to support environmental interventions that may reduce the transmission of oocysts from cattle to humans. This is a particular priority in those populations that are still drinking from untreated surface waters, the bottom ‘rung’ of the WHO/UNICEF Joint Monitoring Program (JMP) ladder (WHO/UNICEF 2017).

In terms of seasonal patterns, a global meta-analysis suggested that in sub-Saharan Africa, cryptosporidiosis peaks follow periods where satellite-derived vegetation indices are low (Jagai et al. 2009). In Kenya nationally, analysis of hospitalised cases suggests that cryptosporidiosis peaks in the driest November–February period (Gatei et al. 2006), whilst in Meru, Kenya, recovery of Cryptosporidium oocysts from surface waters was greatest in the late rainy season and early dry season (Muchiri et al. 2009). However, there is little evidence on how seasonal variation in contact between humans and livestock compares to such seasonal variation in cryptosporidiosis.

Previous studies have used geospatial data to estimate the spatial pattern of cryptosporidium in the landscape at the population level (Burnet et al. 2014; Kato et al. 2004), but this has not been done at the micro scale level using data concerning individual animals. In wildlife ecology, it is common to collect data on individual species via tracking technology such as GPS collars and radio telemetry (Naidoo et al. 2012; Trivelpiece et al. 1986). However, to date, there have been no such individual-level studies that have applied this technology to ‘one health’ problems that entail pathogen movement between livestock and people via the environment.

In this study, we seek to address this gap by using GPS trackers to quantify the movements of individual cattle in a resource-poor area of western Kenya. We use these data to produce spatial summaries of cattle movements by season, including their interactions with drinking-water sources, household compounds, and other herds. We also present a preliminary model of cryptosporidium deposition in the landscape to inform efforts to prevent cryptosporidium-attributable diarrhoeal disease in humans. The study was a component of the OneHealthWater project, which aims to assess child diarrhoeal disease risks in relation to pathogen transmission pathways from livestock through drinking water.

## Methods

### Study site and population

This study was a component of the OneHealthWater project that took place in ten villages in Siaya County, western Kenya, a subsistence farming rural area. In 2011, an estimated 29% of households in Siaya were using streams, rivers, or dams as their main drinking-water source, with 21% taking 30 min or longer to fetch water. Sixteen percent of households reported practicing open defecation (KNBS 2013). In the former Nyanza province, Kenya, in which Siaya lies, *Cryptosporidium* was the second most common pathogen found in infants after rotavirus, and all-cause MSD mortality was particularly high at 3.5% of cases (Kotloff et al. 2013).

### Study design and sampling procedure

The study population for the project was drawn from 1,800 households participating in linked human health-animal health studies within an ongoing Population-Based Animal Syndromic Surveillance (PBASS) study (Mosites et al. 2016; Thumbi et al. 2015). Eligible households for the OneHealthWater project were those participating in the PBASS study and with children aged 6–59 months as the cohort at greatest risk of diarrhoeal disease. A sample size calculation for the main study was powered to detect differences in the proportion of microbially contaminated drinking water between households that owned cattle versus those that did not. Cattle were chosen as a focus livestock species, given that they are common reservoir for *Cryptosporidium*, particularly *C. parvum*. Based on a type 1 error rate of 0.05, 50% cattle ownership, and a desired power of 0.9, an estimated 196 households were required, which was rounded up to 240 households to allow for refusals and drop-outs. A total of 120 cattle-owning households were selected via simple random sampling, as were 120 households that did not own cattle. In planning the GPS tracking component of the study, we included all 120 cattle-owning households and thus also drew on recommendations that the number of individuals tracked should exceed 75 for complex habitat occupation studies (Lindberg and Walker 2007). We sought to maximise the number of individuals sampled rather than length of tracking period per animal, following recommendations in the wildlife management literature (Girard et al. 2006). We conducted a piloting phase to ensure the smooth running of the GPS tracking system where we tracked two animals at each of the three households visited. As confirmed by previous studies (Moritz et al. 2012), we observed that cattle owned by the same household typically move as a herd. For the rest of the fieldwork, we tracked one animal per household, randomly selecting from those animals aged 1 year or older.

### Primary data collection

**Fig. 1.**
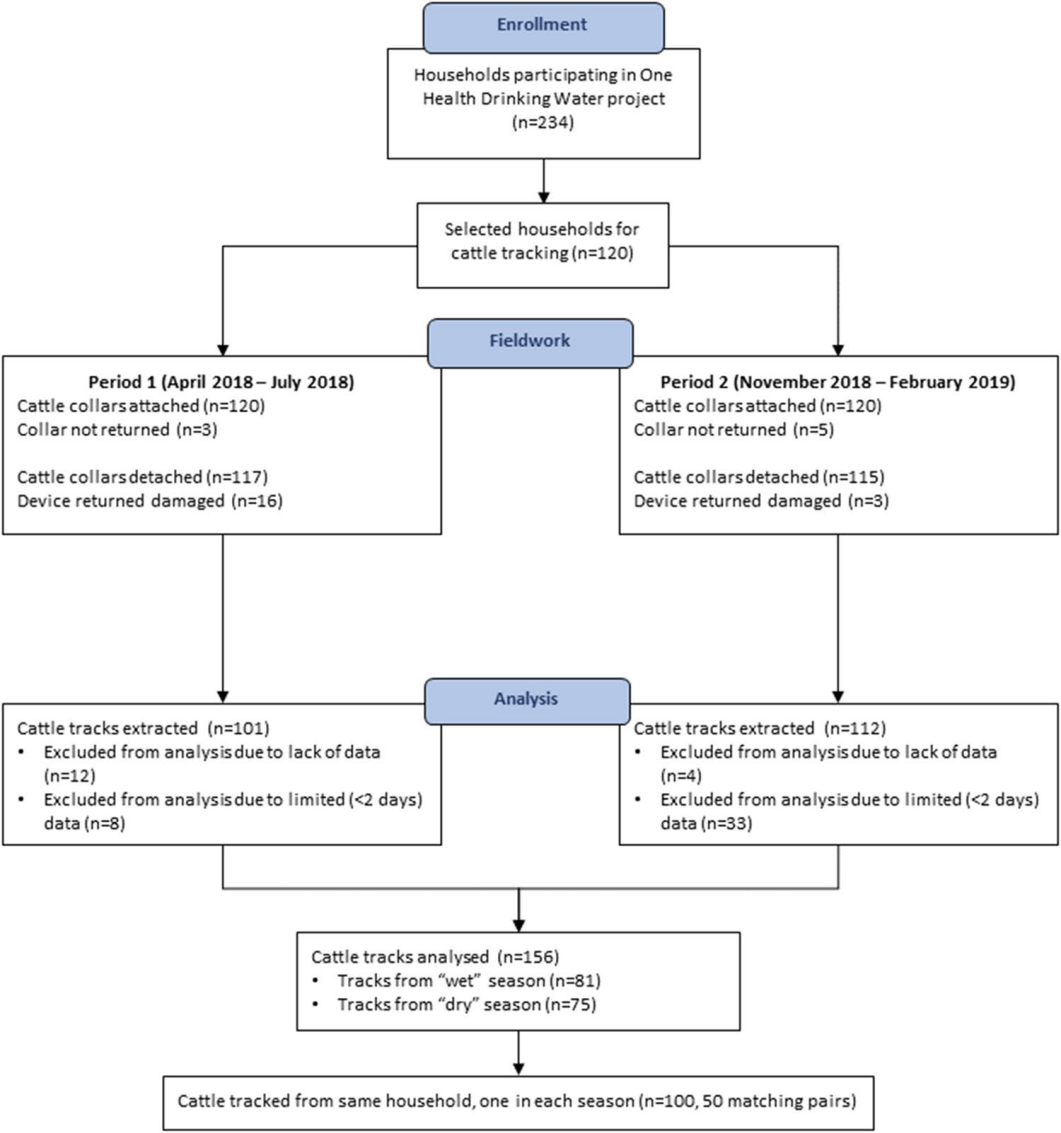
Participant flow diagram for cattle from households selected for GPS tracking.

At each household, the animal to be tracked was selected by assigning each eligible animal in the herd a number, then using a random number generator app on a mobile phone to choose the animal to be tracked. The selected cattle were then fitted with a GPS tracker (Mobile Action i-gotU GT-600 (“Mobile Action Technology Inc., Taipei, Taiwan,” 2018)) attached to an adjustable waterproofed collar, using protocol detailed in a previous study (Floyd et al. 2019). The tracker was set to record precise locational information once every 90 s whilst moving, and to turn off to save battery when stationary. At the end of the week, the researchers returned to the household to collect the trackers and download the data, recording tracker attachment and detachment using electronic forms managed with the CommCare software (http://www.dimagi.com/products/ 2019) on an Android cell phone. Two periods of fieldwork were conducted: one between April and July of 2018, and one between November 2018 and February 2019. Cattle from the same herds were tracked in both periods to capture potential differences in movement patterns and landscape use during different seasons. These seasons were broadly defined as a ‘wet’ season for the period covering the long rains between April and July and a ‘dry’ season for the period spanning the end of the short rains and the beginning of the dry season between November and February according to climate classifications for this region of Kenya (Mugalavai et al. 2008). A participant flow diagram of the cattle data collected is presented in Fig. 1.

For preliminary parameterisation of a spatial faecal deposition model, we also conducted a ‘faecal event’ survey: a team of two researchers observed the daytime behaviour of 33 cattle wearing GPS collars. This survey aimed to measure how often the cattle dropped dung. We repeated the survey once for cattle chosen at random from four household herds, also chosen at random. To mitigate observer fatigue, two researchers took it in turns to observe each animal. Data were recorded via the CommCare software (http://www.dimagi.com/products/ 2019) onto cell phones between 25th January 2019 and 20th February 2019, resulting in 18 days of observation in total.

To map drinking-water sources, gender-balanced discussion groups of between 12 and 18 village residents were convened in each of ten participating villages between 11th July and 17th October 2018. After seeking their informed consent, participants listed the types of water sources that they used, and then mapped the location of each source type via a participatory mapping exercise. Participants recorded water point locations on transparent overlays superimposed on hardcopy maps between 1:4,000 and 1:10,000 scale created with WorldView 2 basemap imagery. Water points recorded were subsequently digitised.

### Data cleaning and movement metric calculations

GPS data from the cattle were cleaned and analysed in R statistical computing version 3.4 (R Core Team 2017) and mapped in QGIS version 3.2.3 (QGIS Development Team 2019). Data from the GPS trackers were first trimmed to delete positional fixes from before and after the collars were attached to the cattle. Erroneous points in the data were removed using the *speedfilter* function from the *trip* package (Sumner 2016) in R. Points that suggested movement at speeds of more than 10 km/h were removed. We used a linear interpolation algorithm to fill in times when no points were collected, giving us datasets with one point per minute for the duration of the tracking time. We then filtered the tracks by hour of the day, to give us daytime (between 7 a.m. and 7 p.m.) movements only and used these datasets for the analysis. We used the *adehabitatHR* package (Calenge 2006) in R to generate gridded Brownian bridge kernel densities for the cattle tracks and 90% volumetric contours. In this package, we used the *kernelbb* function, which required the input of two smoothing parameters. The appropriate value for the first of these, *sig1*, was estimated using the maximum likelihood function *liker*. The second smoothing parameter, *sig2*, was set to be equal to the stated positional accuracy of the GPS device (25 m (“Mobile Action Technology Inc., Taipei, Taiwan,” 2018)).

We calculated a total of six movement metrics for the cattle tracks; three of these were chosen for comparability with other studies of African cattle (Butt 2010a, b; Zengeya et al. 2011). These were the areas of the home ranges from the 90% utilisation distributions, the mean daily distance travelled, and the maximum distance travelled from the homestead, which we calculated using the cleaned GPS data and data on household location recorded via smartphone GPS functionality.

The other three metrics were of direct and indirect contact between cattle and household members, specifically: time spent at water sources, the time spent tethered at household compounds, and the proportion of home range overlap between different herds. To calculate the time each animal spent at drinking-water sources, we first clipped kernel density surfaces to the areas mapped through the participatory workshops. We excluded any piped and rainwater sources mapped by communities, leaving unprotected wells, springs, and surface water points. We then calculated each animal’s time spent at these water point locations as a proportion of the clipped kernel density surface. The amount of time the cattle spent tethered was estimated using an algorithm that initially labelled each GPS location as tethered if it and the previous three locations were within 10 m of each other. In a second step, GPS locations were reclassified as untethered if they were not within a run of at least 15 min of points tagged as tethered. Thus, the algorithm identifies time periods where the animal is likely to have been tethered for longer than 15 min. Lastly, we calculated the Utilisation Distribution Overlap Index (UDOI) for cattle home ranges (Fieberg and Kochanny 2005) using the *kerneloverlaphr* function from the *adehabitathr* (Calenge 2006) package.

We examined the differences in these six movement metrics between the 50 pairs of cattle we collected from the same household over the wet and dry seasons. We used a Wilcoxon signed-rank test to test for significant differences in the mean movement metrics between the two seasons. Finally, we explored the relationship between home range size across the two seasons and home range overlap for the same herds between successive visits. We also tested for differences in the six movements metrics between pairs of cattle tracks collected in different climatologically defined seasons whereby each week of cattle tracking is individually classified as wet or dry based on data from the Climate Hazards Group InfraRed Precipitation with Station (CHIRPS). The results of these analyses are given in the Supplemental materials S1.

### Preliminary faecal deposition model

On preliminary examination, dung deposition counts per period were found to be under-dispersed relative to a Poisson distribution. To account for under-dispersion, we attempted to model dung deposition using a generalised Poisson regression model (Harris et al. 2012) and a two-parameter gamma count model (Sellers and Morris 2017), but neither model converged successfully during fitting. Therefore, despite under-dispersion, a Poisson regression model in Stata version 16 (StataCorp 2019) was used to examine dung deposition rates, using robust regression to account for clustering of observed dung deposition within individual animals. Dung deposition rates were examined in relation to cattle behaviour and animal age/sex, for tethered versus untethered animals, and (as a data quality check) for the two observers.

Finally, to develop a preliminary faecal deposition model, we combined the ‘faecal event’ survey and GPS collar data. The total amount of time tracked cattle spent in each 5 × 5 m pixel was calculated via kernel density analysis of the gap-filled GPS positional fixes. We then generated random numbers from a Poisson distribution across this grid via ArcGIS version 10.7, deriving the average number of events from the ‘faecal event’ survey. We multiplied these two surfaces to estimate dung deposition events, applying published estimates (Lekasi et al. 2001) of faecal wet matter deposition for Kenyan cattle to convert these to kg of wet faecal matter (see Supplemental Materials S2).

## Results

### Seasonal variation in cattle movement

GPS data were successfully collected from 81 cattle in the first period of fieldwork and from 75 cattle in the second period of fieldwork, giving a total of 156 unique GPS tracks. Of the viable tracks, 3 were for calves (< 12 months old), 21 for heifers and 18 for bullocks (both 1–2 years), 20 for adult bulls, and 94 for adult cows. Wilcoxon signed-rank tests for differences between the movement metrics revealed no significant differences between the 46 pairs of cattle from the same households in different seasons as defined by our climatological classification (see Supplemental Materials S1).

**Fig. 2.**
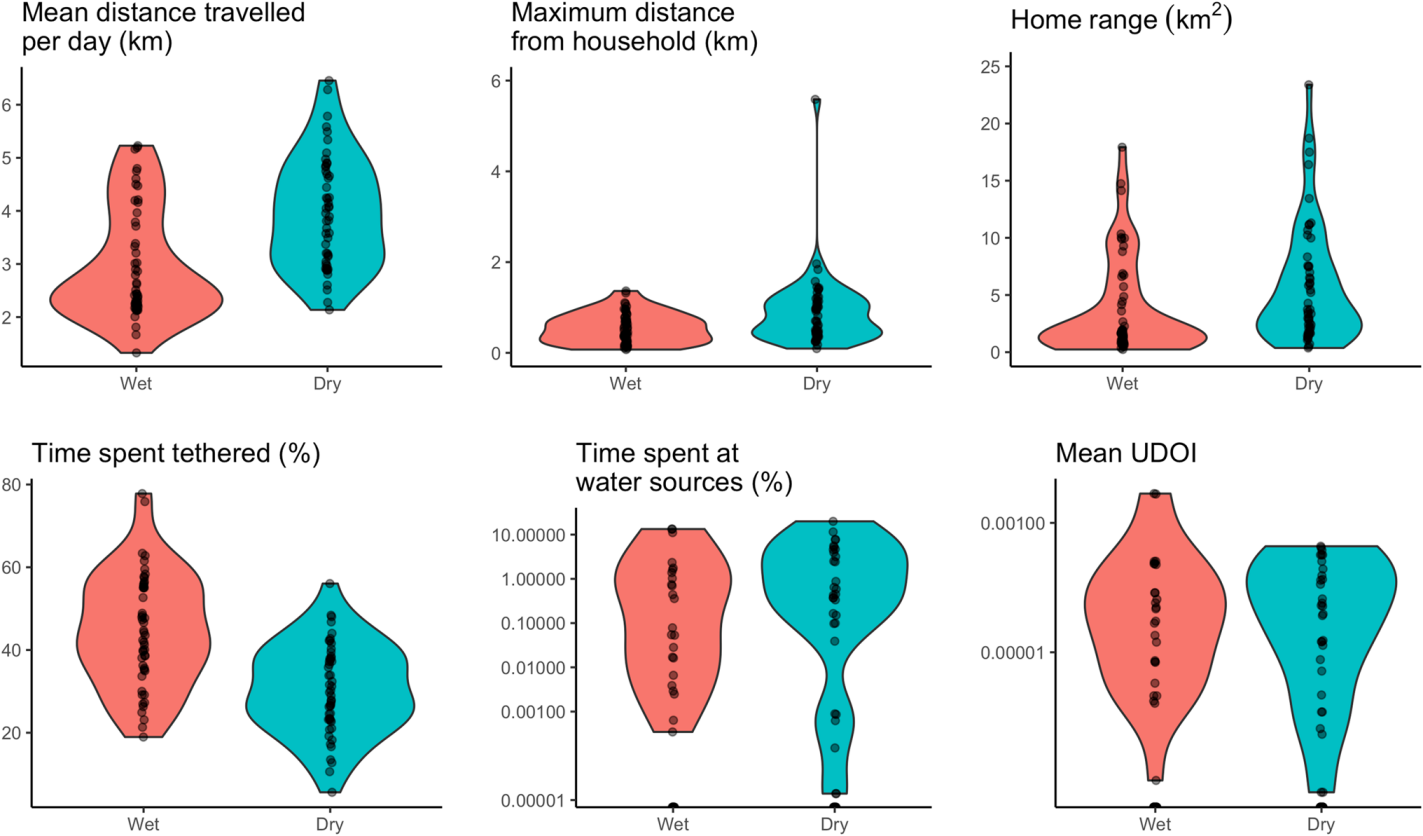
Violin plots showing distributions of cattle movement metrics in the two seasons (*n* = 50 pairs). The width of the violin plot indicates the probability density of the data at different values. UDOI, Utilisation Distribution Overlap Index.

Table 1 summarises 6 movement metrics calculated for the 50 pairs of cattle from the same households collected in different seasons as defined by the different periods of fieldwork. Figure 2 shows the distributions of these 6 metrics across the two seasons where fieldwork was conducted. Overall, we found some significant differences in the movements of cattle between the two seasons, with cattle travelling further, having larger home ranges, and spending less time tethered in the dry season compared to the wet.

**Table 1:** Movement metrics for the 50 pairs of cattle tracked from the same household in different seasons using GPS devices. *UDOI*, Utilisation Distribution Overlap Index.

	Wet season (*n* = 50)		Dry season (*n* = 50)		Paired samples Wilcoxon test (*n* = 50 pairs)
Movement metric	Mean	Standard deviation	Mean	Standard deviation	*p*-value
Total distance travelled from household (km/day)	2.99	*1.05*	3.97	*1.04*	< 0.001***
Maximum distance travelled from household (km)	0.56	*0.33*	0.91	*0.81*	0.003**
Home range (km^2^)	3.78	*4.28*	5.85	*5.21*	0.003**
Time spent tethered (% of daytime)	44.57	*13.64*	30.50	*10.82*	< 0.001***
Time spent at drinking-water points (%)	1.47	*3.64*	2.14	*4.01*	0.265
Home range overlap with other tracked herds (mean UDOI)	1.4710^−4^	5.55*10^*−4*^	7.1*10^−5^	1.24*10^*−4*^	0.334

**Fig. 3.**
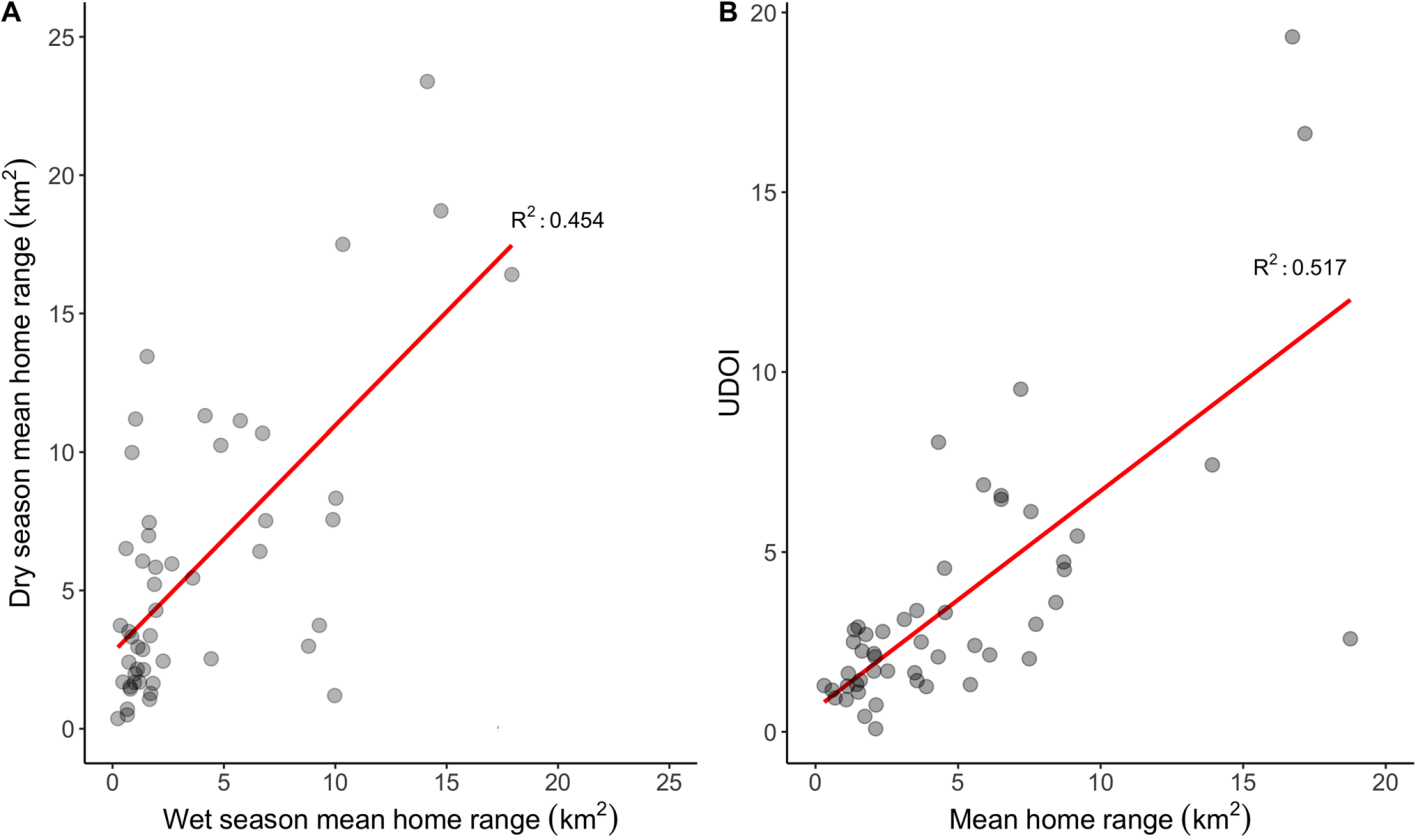
**A** Relationship between the size of home range areas in the two seasons for pairs of cattle (*n* = 50 pairs). **B** Relationship between size of home range area (mean across the two seasons where fieldwork was conducted) and Utilisation Distribution Overlap Index (UDOI) for pairs of cattle (*n* = 50 pairs). Red lines are regression lines. A high UDOI indicates a high degree of overlap.

We examined the relationship between the home range areas across the two seasons and fitted a linear regression model to the data from 50 pairs of cattle, with an *R*^2^ of 0.454 (Fig. 3A). We also explored the relationship between home range area and the UDOI. Higher UDOIs are indicative of higher degrees of overlap between home ranges. A UDOI of over 1 indicates utilisation distributions that are non-uniformly distributed and have a high degree of overlap. We found a positive relationship between home range size and UDOIs for the pairs of cattle tracked from the same household (Fig. 3B). Although home ranges were higher overall during the dry season, the high degree of overlap between the utilisation distributions and correlation with home range areas from the wet season suggests that landscape usage by the cattle was similar across the two seasons.

### Preliminary spatial model of faecal deposition by cattle

Table 2 summarises the characteristics of cattle and their behaviours observed through the survey. In total, 140 dung deposition events were observed over 185.4 h, giving a mean deposition rate of 0.76 stools/h. Thirty-three animals were observed in total, comprising eight heifers, three bullocks, two bulls, and 20 cows. All animals were observed moving, drinking, and grazing; all bar two were observed standing or lying; and 13 were tethered for part or all of the observation period.

**Table 2:** Cattle characteristics and behaviour during dung deposition survey

	Observation time — hours (%)
Cattle behaviour:	
Moving	139.8 (75.4%)
Grazing	29.8 (16.1%)
Standing/sitting	31.3 (16.9%)
Standing in water/drinking	1.6 (0.9%)
Cattle demographic:	
Heifers (1–2 years)	47.6 (25.7%)
Bullocks (1–2 years)	17.0 (9.1%)
Bulls (> 2 years)	10.0 (5.4%)
Cows (> 2 years)	110.8 (59.8%)
Time tethered	73.9 (39.8%)
Total	185.4

Table 3 shows dung deposition rate ratios, derived through Poisson regression modelling. Bulls defecated significantly more often than other cattle (incidence rate ratio: 1.29). None of the other covariates examined was significantly associated with the observed dung deposition rate, though the dung deposition rate was 2.47 times higher when livestock were stood in or drinking water.

**Table 3:** Dung deposition rate ratios by observer identity, cattle activity, and demographic types, as estimated through univariate Poisson regression models

	Dung deposition rate ratio (95% confidence intervals)	*p* value
Cattle activity:		
Grazing	0.65 (0.37 to 1.14)	0.14
Moving	1.19 (0.81 to 1.75)	0.37
Standing/sitting/lying	0.83 (0.45 to 1.55)	0.56
Standing in water/drinking	2.47 (0.75 to 8.14)	0.14
Cattle demographic (reference: heifers)		
Bullocks 1–2 years	0.94 (0.64 to 1.37)	0.73
Bulls > 2 years	1.29 (1.03 to 1.62)	0.03
Cows > 2 years	1.13 (0.86 to 1.49)	0.38
Tethered	1.27 (0.96 to 1.69)	0.10
Recorded by 2nd observer	1.14 (0.86 to 1.51)	0.37

**Fig. 4:**
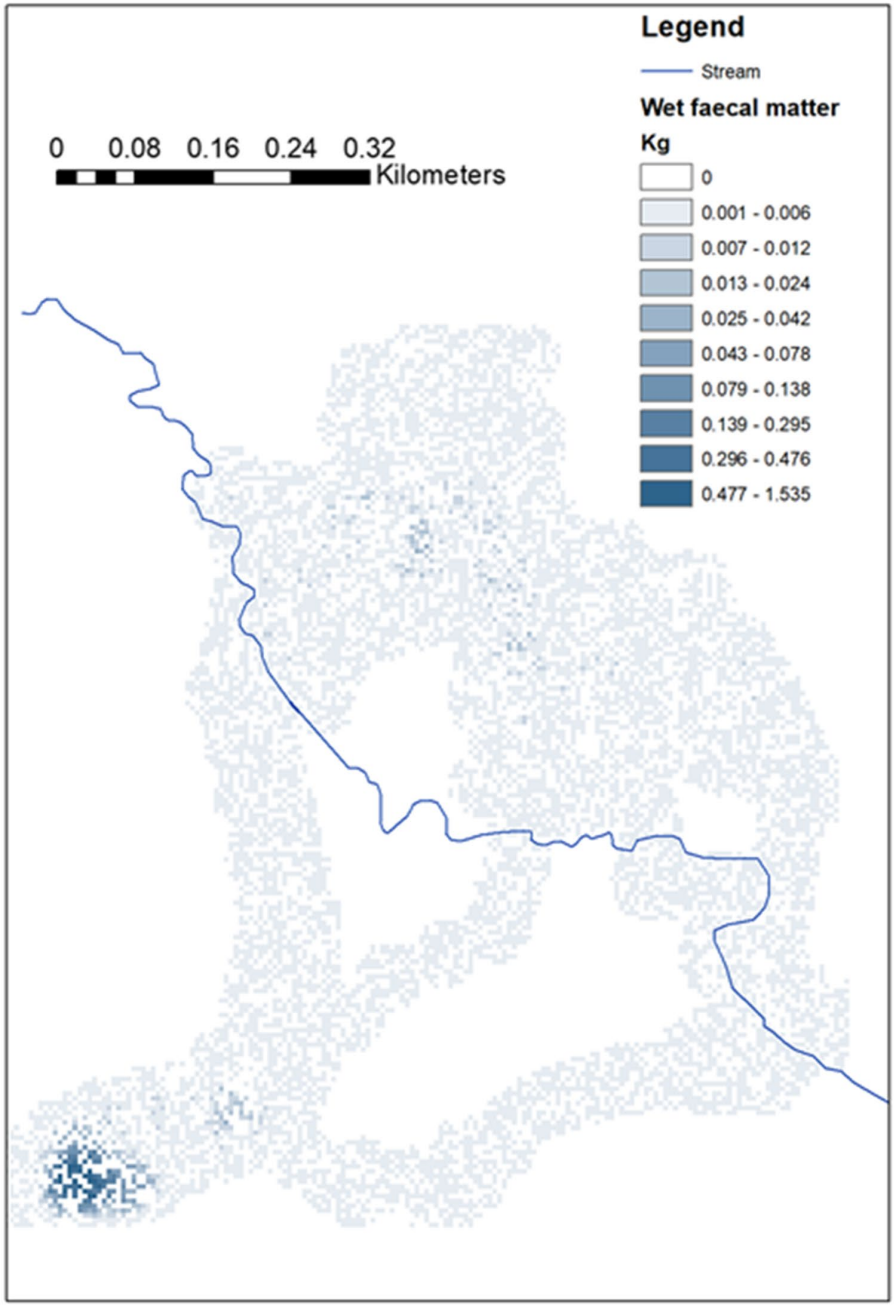
Modelled wet matter faeces deposited per 5 × 5 m pixel by an example adult cow over 9 days of observation

Figure 4 shows the modelled wet matter faecal deposition by an example adult cow, tracked over 9 days. As dung deposition did not vary significantly by activity (Table 3), it was held constant in modelling. In Fig. 4, there are high levels of dung deposition in the southwest where the animal is tethered at night, and somewhat higher deposition in grazing lands north of the stream, with herding routes to the grazing areas having lower dung deposition.

## Discussion

Our study is the first to quantify seasonal variation in the time that livestock spend at water sources used for domestic water supply. Our findings show that in both seasons studied here, cattle consistently come into direct contact with surface water sources used by people and are generally tethered close to the home. We observed significant seasonal differences in movement patterns, with cattle having higher home ranges, travelling further, and spending less time tethered in the dry season. However, in this population, we observed no significant seasonal variation in the time cattle spend close to water sources, and we found high degrees of overlap in the home ranges of cattle measured on successive visits. Seasonal variation in diarrhoeal diseases such as cryptosporidiosis is thus more likely to be driven by factors other than seasonal herding patterns. Since domestic rainwater harvesting is widespread (Okotto-Okotto et al. 2019), when rainwater harvesting is not an option in the dry season, some households switch drinking-water sources from rainwater to sharing surface waters with cattle. This seasonal change in human behaviour rather than cattle herding is consistent with seasonal diarrhoea variation. Environmental transport of cattle faeces and associated pathogens may also vary seasonally, for example as oocysts are transported by wet season run-off.

Relative to our study population, other cattle herds studied in arid and semi-arid East Africa travel further, have larger home ranges, and exhibit greater seasonal variation in movement. For example, relative to the mean maximum distances of less than 1 km travelled by cattle in our study in both wet and dry seasons, mean maximum distances travelled by herds owned by Maasai pastoralists in Narok County, Kenya, were 3.6 km in the wet season, 3.9 km in the dry season, and 7.6 km in the drought season (Butt 2010a). Whilst seasonal cattle movements do not account for seasonal diarrhoea incidence variation in Siaya County, they may be significant in semi-arid and arid pastoralist areas elsewhere. Whilst other studies (Butt 2010b; Zengeya et al. 2011) have quantified the time cattle spend at water sources, they have not differentiated water sources shared with people from those used exclusively by cattle. By combining GPS track data from livestock with a water point mapping database, we were able to quantify such contact by season and thereby one component of waterborne zoonotic disease transmission risk. In measuring home ranges, we further developed the movement metrics of these earlier studies by incorporating a Brownian Bridge model into our methodology. Unlike earlier studies which are only based on positional fixes, this approach is becoming more widespread in wildlife ecology (Walter et al. 2015) because it takes account of temporal animal movement patterns in generating kernel density surfaces.

A recent systematic review (Malan et al. 2018) suggests that environmental interventions to separate livestock from water may be more effective in the tropics than in colder climates. However, whilst GPS devices have been used to evaluate the impact of providing off stream watering points on cattle movement in Australia (Kaucner et al. 2013), no equivalent evidence on such interventions is yet available from developing countries. Environmental loadings of *Cryptosporidium* and *Giardia* from livestock have been quantified at district level in India (Daniels et al. 2015), but not at a more detailed, landscape scale. Building on this district-scale work, our preliminary cattle faecal deposition model provides insights into how livestock deposit faeces at the landscape scale in rural Kenya, suggesting direct deposition into water sources used by people. The higher dung deposition rate for animals stood in water (Table 3) is consistent with informal feedback from local herders, as is greater deposition by bulls.

In future, through more comprehensive data collection and modelling, it would be possible to model pathogen deposition patterns from deposited faecal matter. This would require enumeration of *Cryptosporidium* oocyst shedding by Siaya’s cattle population, but at present, such data are only available for other East African cattle populations (Nizeyi et al. 2002). A more fully developed model would need to consider environmental transport of oocysts (e.g. through run-off modelling) and not solely their deposition. There would also be scope to generalise grazing behaviour beyond the sampled herds through agent-based modelling, which has been used to simulate cattle grazing for other purposes (Jablonski et al. 2018). Although we did not observe collection of animal dung for manure, flooring construction, or fuel during fieldwork, such economic uses of cattle faeces do occur elsewhere in Kenya (Lekasi et al. 2001), so would require quantification in some settings.

Given growing interest in building evidence concerning ‘One Health’ interventions for safe separation of livestock and people in the environment (Penakalapati et al. 2017), further development of such livestock movement models and GPS tracking technology could prove valuable. Firstly, intervention evaluation studies are known to be costly and need to be underpinned by sufficient preliminary evidence of their potential effectiveness. A livestock movement model could inform such an evidence base, concerning the impacts of candidate interventions such as fencing off domestic water sources from livestock (Penakalapati et al. 2017). Secondly, GPS tracking technology could be a valuable intervention evaluation tool. Not only would it enable measurement of an intermediate outcome, namely a reduction in human-livestock contacts and thereby monitor intervention compliance, but it could also provide insights into unintended harms from such interventions. Taking the example of fencing domestic water sources off from livestock, such potential harms could include disruption to livestock herding and water access with implications for livestock welfare, milk production, and thereby child health. Further development of GPS tracking technology and related models therefore seems justified.

Our findings are subject to several limitations. Firstly, loss or malfunctioning of some GPS devices meant we were only able to track a subset of cattle in our sample. If systematic differences exist between herds with and without functioning GPS devices or between households participating in the PBASS study and the wider cattle-owning population, this could introduce bias into our findings. Herders could also have modified their cattle management practices in the knowledge that their livestock were being monitored. As with other similar studies (Kaucner et al. 2013; Zengeya et al. 2011), although this is mitigated by frequent positional fixes for tracked cattle, our estimates of time cattle spend at water sources are subject to the positional accuracy of GPS devices used to map water points and record cattle movement. They are thus sensitive to the kernel bandwidth used in post-processing of GPS locations from collars. Our pilot faecal event survey also had a comparatively small sample size, and so had little statistical power to detect differences in faecal deposition rates between activities and cattle demographic groups. Moreover, the faecal event survey was only undertaken in one of the two seasons studied, meaning we were not able to compare differences in faecal deposition events across seasons. Whilst we found no effect of observer identity on mean faecal deposition counts (Table 3), under-dispersion in count data is rare and could suggest problems with the data recording protocol (Sellers and Morris 2017).

In conclusion, the findings presented here demonstrate the feasibility and value of GPS tracking to examine seasonal differences in livestock movements, and have revealed some important differences in movement patterns between the seasons studied. Although cattle movements are unlikely to be driving seasonal patterns in diarrhoeal diseases in this setting, the direct deposition of cattle faeces into water sources used by people highlights how further work to model deposition in the landscape could inform interventions to mitigate this disease transmission pathway in similar settings, thereby reducing the incidence of diarrheal diseases and improving population health.

## Supplementary Information

ESM 1 (DOCX 22 kb)
