# Supplementary material for: A spatiotemporal analysis of cattle herd movement in relation to drinking-water sources: implications for Cryptosporidium control in rural Kenya
Source: Environ Sci Pollut Res Int. 2022 Jan 17;29(23):34314–24. doi: 10.1007/s11356-021-17888-3 (PMC9076705; doi:10.1007/s11356-021-17888-3)
Supplement: Supplementary file 1 — (DOCX 22 kb) [file 11356_2021_17888_MOESM1_ESM.docx]

# Supplemental materials for the manuscript “A spatiotemporal analysis of cattle herd movement in relation to drinking-water sources: implications for Cryptosporidium control in rural Kenya”

## S1 Seasonal differences as defined by CHIRPS data

To identify wet and dry seasons in the time periods when the cattle were tracked, ten years of daily rainfall data (from 1^st^ Jan 2008 to 31^st^ Dec 2017) derived from the Climate Hazards Group InfraRed Precipitation with Station (CHIRPS) version 2 dataset (Funk et al., 2015) at 0.05 x 0.05 degree (~5km) spatial resolution were used to define climatological wet and dry seasons at a locational level. Each week of cattle tracking was subsequently classified as either ‘wet’ or ‘dry’ according to these definitions. Wilcoxon signed-rank tests for differences between the movement metrics across these seasons revealed no significant differences between the 46 pairs of cattle from the same households in different seasons as defined by our climatological classification (Table S2).

Table S1 Movement metrics for the 46 pairs of cattle tracked across climatologically-defined seasons using GPS devices.

|  | Dry season | Wet season | Paired Samples Wilcoxon Test (n = 46 pairs) |
| --- | --- | --- | --- |
| **Movement metric** | **Mean (standard deviation)** | **Mean (standard deviation)** | **Value (p)** |
| Total distance travelled from household (km/day) | 3.56 (1.21) | 3.59 (1.35) | 0.6136 |
| Maximum distance travelled from household (km) | 0.784 (0.424) | 0.678 (0.411) | 0.1375 |
| Home range (km^2^) | 8.70 (6.85) | 8.13 (8.00) | 0.1438 |
| Time spent tethered (% of day-time) | 40.1 (19.3) | 36.5 (16.4) | 0.6947 |
| Time spent at drinking-water points (%) | 0.023 (0.046) | 0.017 (0.039) | 0.477 |
| Home range overlap with other tracked herds (%) | 0.57 (0.23) | 0.68 (0.38) | 0.8326 |

## S2 Estimation of dung deposition events

To develop the preliminary faecal deposition model, we combined data from the ‘faecal event’ survey and GPS collar data. The total amount of time tracked cattle spent in each 5 x 5 metre pixel was calculated via kernel density analysis of the gap-filled GPS positional fixes. We then generated random numbers from a Poisson distribution across this grid via ArcGIS version 10.7, deriving the average number of events from the ‘faecal event’ survey. We multiplied these two surfaces to estimate dung deposition events, applying published estimates of faecal wet matter deposition for Kenyan cattle to convert these to Kg of wet faecal matter (Table S1). Table S1 shows the estimates used to derive the total faecal matter produced per day for each of the different demographic groups of cattle, as well as estimates for the oocysts shed by each class of cattle, although these were not used in the deposition model as further validation would be needed to model this adequately.

Table S2 Theoretical faecal deposition of Cryptosporidium oocysts for different demographic categories of cattle. Data were not available for cattle less than 12 months old.

| Cattle demographic | Mean liveweight(Lukuyu et al., 2016) (kg) | Faecal dry matter produced per day(Lekasi et al., 2001) (g) | Total faecal matter produced per day (g)(Lekasi et al., 2001) | Oocysts/g of faeces (Nizeyi et al., 2002) | Oocysts shed per day |
| --- | --- | --- | --- | --- | --- |
| Heifers (1-2 years) | 195 | 1560 | 3900 | 210 | 8.19 x 10^5^ |
| Bullocks (1-2 years) | 195 | 1560 | 3900 | 210 | 8.19 x 10^5^ |
| Adult bulls (more than 2 years old) | 228 | 1824 | 4560 | 210 | 9.58 x 10^5^ |
| Adult cows (more than 2 years old) | 228 | 1824 | 4560 | 210 | 9.58 x 10^5^ |
